# All That Glitters Is Not Gold" - A Case of an Occult Foreign Body in the Lung with Elevated 2-[18F]-Fluoro-2-deoxy-D-glucose (FDG) Uptake Mimicking Bronchogenic Carcinoma

**DOI:** 10.7759/cureus.990

**Published:** 2017-01-23

**Authors:** Venkatkiran Kanchustambham, Aaron Schenone, Brian A Reichardt, Swetha Saladi, Kris Mehta, Nishant Poddar, David Stoeckel

**Affiliations:** 1 Pulmonary and Critical Care Medicine, Saint Louis University School of Medicine; 2 Internal Medicine, Saint Louis University School of Medicine; 3 Division of Hematology and Oncology, Saint Louis University School of Medicine

**Keywords:** occult foreign body, pet/ct, fdg, foreign body granuloma, foreign body aspiration (fba)

## Abstract

Combined positron emission tomography/computed tomography (PET/CT) using the glucose analogue 2-[18F]-fluoro-2-deoxy-D-glucose (FDG) has become the standard of care in oncological patients. However, due to the non-specific nature of FDG uptake, there are many physiological variants and benign pathological entities that also demonstrate augmented glucose metabolism, such as inflammatory and infective processes. Undiagnosed and retained foreign bodies (occult foreign bodies) in the lung can induce inflammatory reaction consisting of polymorphonuclear neutrophils, macrophages, and granulation tissue resulting in intense FDG uptake because of high metabolic activity and cell turnover. Here, we present a case of an occult foreign body imitating a tumor on PET/CT.

## Introduction

Combined positron emission tomography/computed tomography (PET/CT) using the glucose analogue, 2-[18F]-fluoro-2-deoxy-D-glucose (FDG) has become the standard of care in oncology patients as it has significant impact on tumor staging and re-staging, detection of recurrent disease, and optimization of therapy in a wide variety of malignancies. However, due to the non-specific nature of FDG uptake, there are many physiological variants and benign pathological entities that also demonstrate augmented glucose metabolism, such as inflammatory and infectious processes [1]. Knowledge of these potentially false-positive lesions is essential for accurate PET/CT interpretation in oncology patients.

Even though foreign body aspiration (FBA) is more frequent in infants and small children, it can still occur at any age among adults even in the absence of any predisposing factors. Undiagnosed and retained foreign bodies (occult foreign bodies) in the lung can induce an inflammatory reaction consisting of polymorphonuclear neutrophils, macrophages, and granulation tissue, resulting in an intense FDG uptake because of high metabolic activity and cell turnover, which can form a mass mimicking soft tissue tumors that may be FDG avid and lead to a diagnostic dilemma [2-3]. Here, we present a case of an occult foreign body imitating tumor on PET/CT.

## Case presentation

A 25-year-old Caucasian male with no significant past medical history was admitted to the pulmonary service for further workup of hemoptysis in May of 2015. The patient initially presented to an outside hospital in 2014 with three episodes of frank hemoptysis that was two to three tablespoons in quantity. It was associated with shortness of breath on exertion and cough that was productive with yellowish sputum. During that admission, he underwent a CT scan of the chest that showed a 8 cm lobulated mass in right lower lobe with right hilar nodes. He underwent bronchoscopy with mediastinoscopy due to the concern for malignancy. There were no endobronchial lesions seen per the report and pathology showed squamous metaplasia with ulceration. He left against medical advice (LMA) prior to completion of further workup.

The patient was seen in our emergency room in May 2015 with a cough with blood-tinged sputum that has gotten worse since his previous admission. He also endorsed 10-pound weight loss and poor appetite. He denied any fever, chills, foul smelling sputum, or recent travel outside the country. He was currently unemployed and smoked one pack of cigarettes daily for last five years. There was no significant family history.

**Figure 1:**
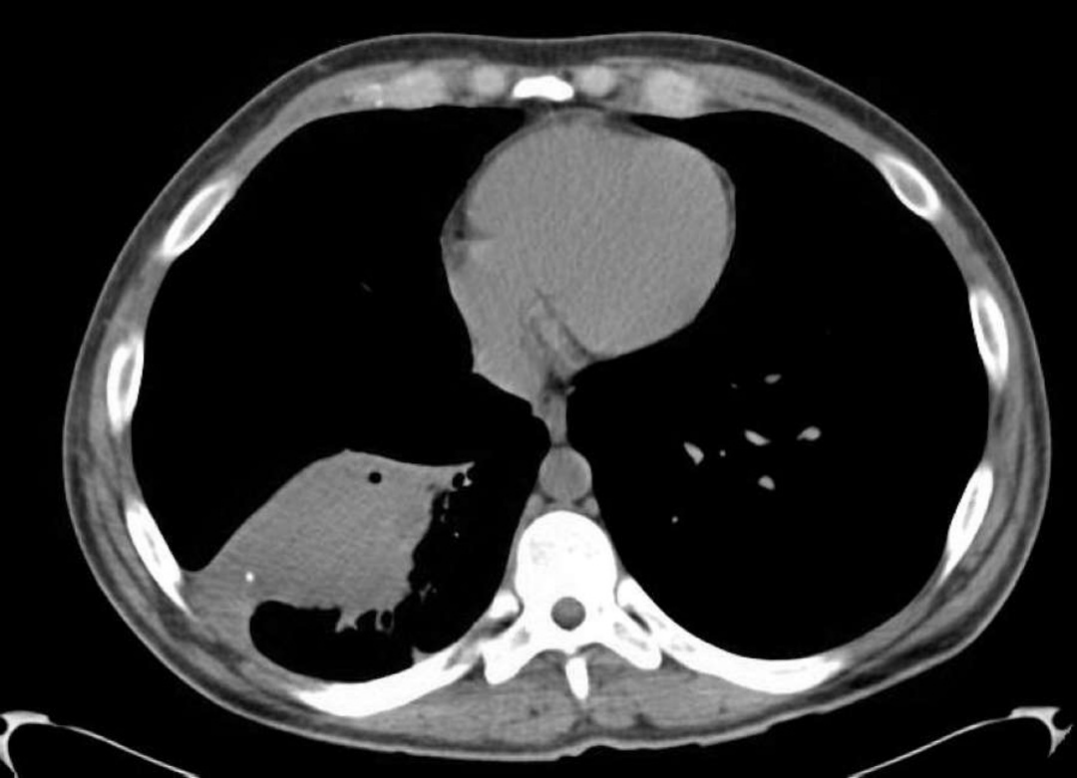
Mediastinal window of the chest CT. Irregular mass-like consolidation in the right lower lobe abutting the major fissure and diaphragmatic pleura, measuring at least 9.5 x 5.0 cm

**Figure 2:**
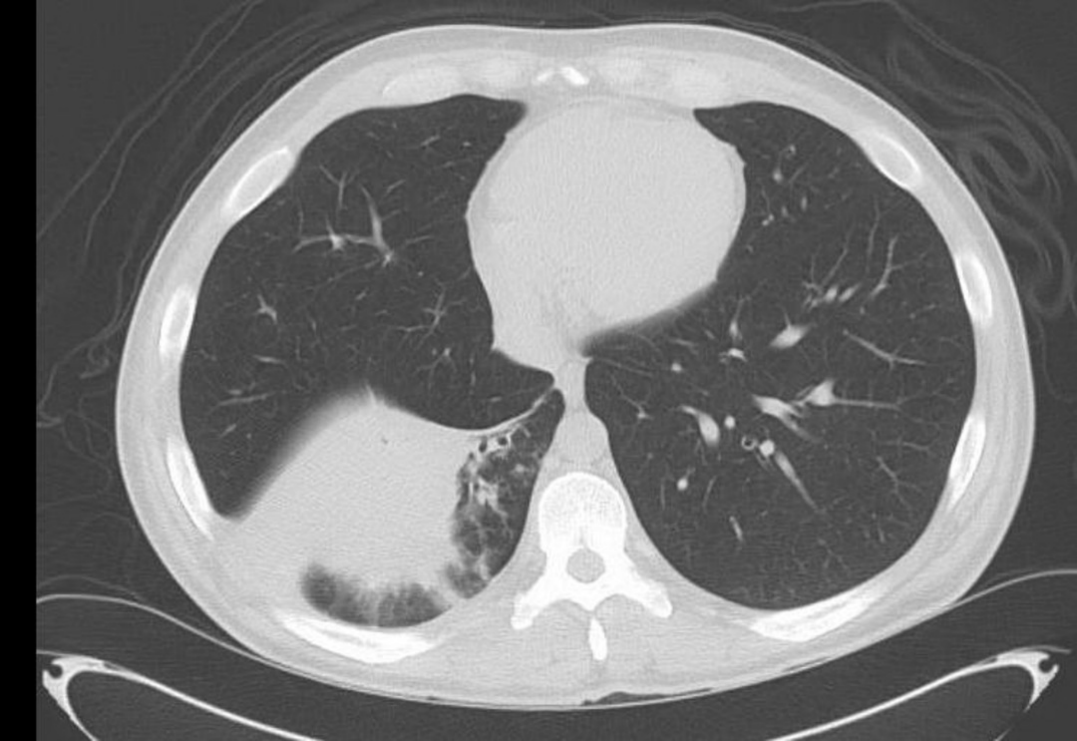
Lung parenchyma window. Mass-like consolidation in the right lower lobe with surrounding bronchiectasis

**Figure 3:**
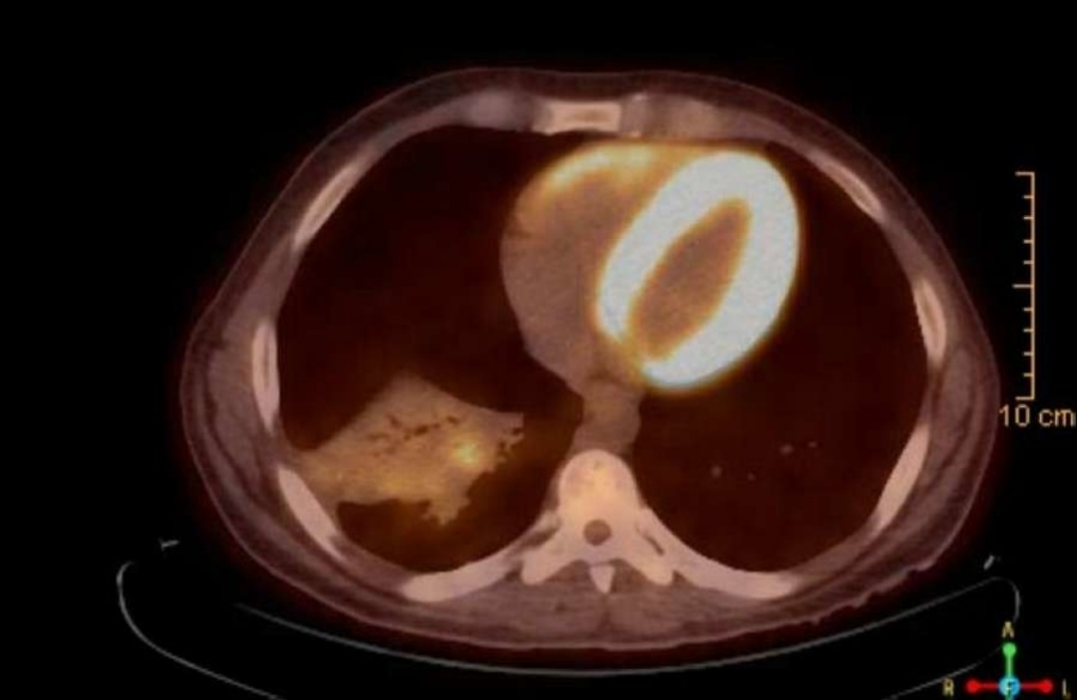
Ill-defined FDG-avid mass measuring 9.5 x 5.0 cm involving the right lower lobe demonstrating a standardized uptake value (SUV) max of 4.5 on PET/CT, concerning for malignancy. FDG: 2-[18F]-fluoro-2-deoxy-D-glucose

Informed patient consent was obtained for his treatment. The patient was started on broad-spectrum antibiotics and underwent a CT scan of the chest that showed an irregular mass-like consolidation measuring 9.5 x 5.0 cm in the right lower lobe with adenopathy and right lower lobe bronchiectasis (Figures 1-2). He also underwent a PET/CT that was significant for an FDG-avid mass in the right lower lobe demonstrating an standardized uptake value (SUV) max of 4.5 (Figure 3).

**Figure 4:**
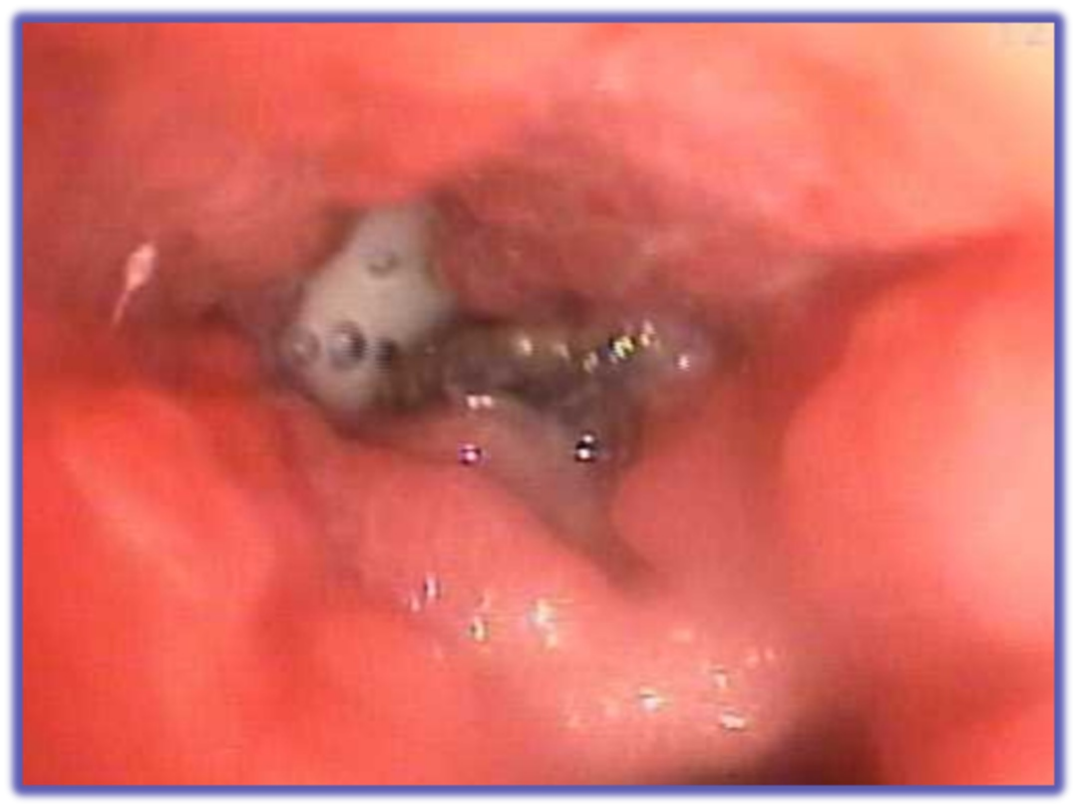
Bronchoscopy that was remarkable for copious purulent secretions in all large airways with friable mucosa

**Figure 5:**
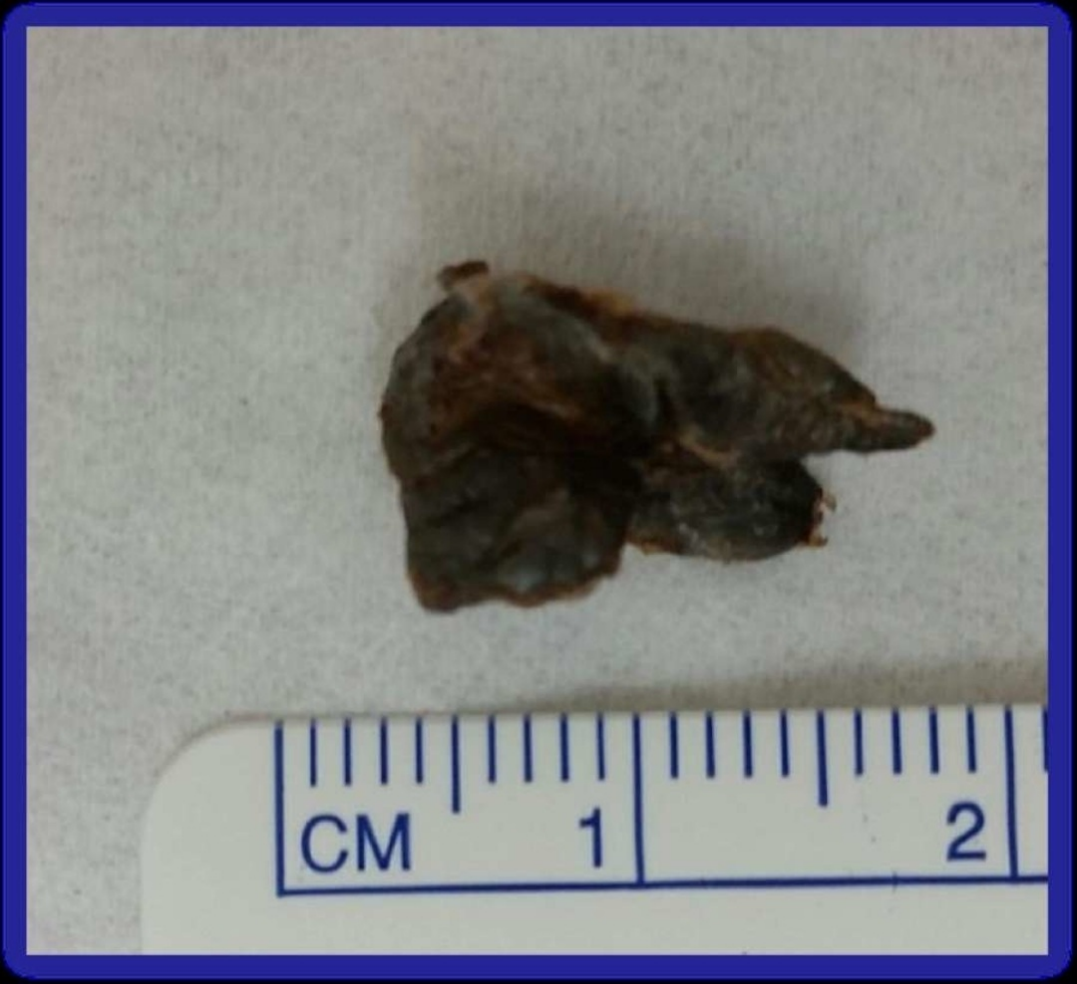
Irregularly shaped 1.5 x 1.0 x 0.4 cm green-gray flexible floating material found to be non-viable foreign material

The patient underwent flexible bronchoscopy that was remarkable for copious purulent secretions in all large airways with friable mucosa (Figure 4) and marked mucosal irregularities in the right lower basal segments. Once the secretions were cleared, a gray-colored solid foreign body (approximately 1.0 x 0.5 cm) in right lower lobe was seen that was removed with toothed forceps (Figure 5). Pathology was remarkable for non-viable foreign material and underlying submucosa with diffuse lymphoplasmacytic infiltrate admixed with neutrophils, eosinophils, and a few granulomas with no malignancy features. 

On repeated questioning, the patient admitted to habitual chewing of plastic objects and recalled one episode in 2009 when he may have fallen asleep while chewing on a plastic object. He was given antibiotics for two weeks and was discharged home with a plan to repeat a CT scan in six to eight weeks. However, he was lost to follow-up again. We hypothesize that this episode in 2009 may have caused the unintentional aspiration of the foreign body into the lungs, which the patient was unaware of. This occult foreign body caused foreign body granuloma resulting in a tumor-like mass with intense FDG avidity mimicking malignancy.

## Discussion

FDG is a non-physiological analogue of glucose that, once injected, is taken up by cell membrane glucose receptors mainly, the glucose transporter-1 (GLUT-1) molecule, which transports it into the intracellular compartment where it is phosphorylated into FDG-6-phosphate by the enzymatic action of hexokinase. Cancer cells show increased uptake of glucose (due to an overexpression of glucose transporter proteins) and an increased rate of glycolysis. However, following phosphorylation by hexokinase, FDG becomes trapped in the cancer cell, failing to undergo further metabolism due to down-regulation of phosphatase [1].

Inflammation is the primary response of the lung immune system to infection or irritation, such as an occult foreign body. Activated white blood cells (granulocytes, lymphocytes, and macrophages), which have enhanced levels of glucose transporters, especially GLUT 3 as well as increased affinity to 18F-FDG through various cytokines and growth factors, are recruited. These pathophysiologic processes are the basis for increased uptake of FDG that is encountered in inflammatory processes [1-2].

The inflammatory reaction induced by retained foreign body can be an exudative reaction, leading to the secondary infection with abscess formation or an aseptic fibrinous response, resulting in the development of a foreign body granuloma. When the retained foreign body cannot be digested or degraded by the neutrophils and macrophages, they fuse together forming foreign body giant cells (FBGCs) and foreign body granuloma. FBGCs have an enhanced hypermetabolic state, resulting in an increased localized uptake of FDG and can cause a false-positive PET/CT [1-2].

The patient presented with radiological evidence of a lung mass; however, after bronchoscopic examination, he proved to have a plastic foreign body in the bronchus, which was retrieved under general anesthesia using a flexible bronchoscopy. On repeated questioning, he reported habitual chewing of plastic objects and recalled falling asleep while chewing four to five years prior to the current admission.

In a retrospective study evaluating the natural course of FBA in adults, patients were divided into acute and chronic groups based on the time of presentation. In the chronic group, the mean duration, before the foreign body was diagnosed, was 25 months. The chronic group did not have a choking history at the time of presentation, hence, delaying the diagnosis with a resultant occult or retained foreign body, as in this case [3].

There are numerous case reports of FBA in the lung mimicking lung cancer; however, not many of them had a PET/CT as part of the workup to show an increased FDG avidity of the lesions. Two case reports of positive PET/CT findings of foreign body granulomas in the lungs by retained textiles are described by García de Llanos, et al. and Mouroux, et al. [4-5]. Both cases were due to retained surgical sponge material. Ruiz-Zafra, et al. described two cases of surgical adhesive, which are frequently used after pulmonary resection to prevent or reduce pulmonary air leakages, as the cause of the false-positive PET/CT. Subsequent surgical exploration showed that the lesions were foreign body reactions to the bioadhesive [6]. Yüksel, et al. and Lim, et al. described a total of five cases of PET/CT-positive inflammatory nodules that developed secondary to suture or stapler material granulomas [7-8]. 

In regards to an occult foreign body in the lungs causing a positive PET/CT, we have come across two case reports in the literature [9-10]. One was an abstract that described increased FDG due to the impacted foreign body that was subsequently removed through bronchoscopy and was proven to be a fish bone [9]. The patient was 62 years old and presented with a recurrent cough. The second case described PET/CT-positive right lung nodules in a patient with a history of left lung cancer post-resection [10]. A biopsy was performed, which confirmed the nodule to be a granuloma with foreign body giant cells and debris of calcifications. The patient was 49 years old and asymptomatic at the time of presentation. In our case, the patient presented with hemoptysis and was only 25 years old at the time of presentation.

## Conclusions

To conclude, an accurate interpretation of PET/CT findings requires knowledge of the normal physiologic distribution of FDG and potential pitfalls due to benign conditions that are PET-positive. Awareness of these potential pitfalls is important in preventing misinterpretation of false-positive PET/CT findings in the thorax. The current case shows the importance of suspecting a tracheobronchial FBA in the differential diagnosis of mass-like lesions on the CT chest with positive PET/CT or unexplained chronic respiratory symptoms and emphasizes the importance of detailed history-taking.
